# ﻿Four new araneogenous species and a new genus in Hypocreales (Clavicipitaceae, Cordycipitaceae) from the karst region of China

**DOI:** 10.3897/mycokeys.112.140799

**Published:** 2025-01-23

**Authors:** Wan-Hao Chen, Dan Li, Hui-Lin Shu, Jian-Dong Liang, Jie-Hong Zhao, Wei-Yi Tian, Yan-Feng Han

**Affiliations:** 1 Center for Mycomedicine Research, Basic Medical School, Guizhou University of Traditional Chinese Medicine, Guiyang 550025, Guizhou Province, China; 2 Institute of Fungus Resources, Department of Ecology, College of Life Sciences, Guizhou University, Guiyang 550025, Guizhou Province, China; 3 Key Laboratory of Microbio and Infectious Disease Prevention & Control in Guizhou Province, Guiyang 550025, Guizhou Province, China; 4 College of Pharmacy, Guizhou University of Traditional Chinese Medicine, Guiyang 550025, Guizhou Province, China

**Keywords:** *Chlorocillium*, lecanicillium-like, morphology, phylogenetic analysis, spider

## Abstract

The karst region in southwestern China is one of the biodiversity hotspots in the world with rich fungal diversity but under-studied. Four fungal species belonging to *Chlorocillium* (Clavicipitaceae) and *Gamszarella* (Cordycipitaceae) were isolated from dead spiders. Morphological comparisons, phylogenetic analyses and a PHI analysis based on multigene datasets support the establishment of these new species viz., *Chlorocilliumguizhouense***sp. nov.**, *C.vallense***sp. nov.**, *Gamszarellasinensis***sp. nov.**, and *G.vallensis***sp. nov.** are introduced. A new genus, *Neogamszarella*, is proposed to accommodate *Gamszarellaantillana*, which is phylogenetically distinct from *Gamszarellas. str.* Our results revealed that further attention needs to be paid to the diversity of araneogenous fungi in the karst regions of southwestern China.

## ﻿Introduction

Araneogenous or araneopathogenic fungi are spider-pathogenic fungi and are one of the ecologically important groups of fungi (Evans and Samson 1987). Formerly, araneogenous fungi were restricted to three families in Hypocreales: Cordycipitaceae (Kobayasi and Shimizu 1982; Chen et al. 2018; Mongkolsamrit et al. 2018) and Ophiocordycipitaceae (Kobayasi 1941; Samson and Evans 1975), and one species in Bionectriaceae (Chen et al. 2016a; Shrestha et al. 2019). However, Chen et al. (2022a) reported two new spider-associated genera in Clavicipitaceae. Wang et al. (2024a) reported *Akanthomyces* Lebert, *Beauveria* Vuill., *Cordyceps* Fr., *Engyodontium* de Hoog, *Gibellula* Cavara, *Hevansia* Luangsa-ard et al., *Lecanicillium* W. Gams & Zare, *Samsoniella* Mongkols. et al., *Torrubiella* Boud., *Jenniferia* Mongkols. et al., *Polystromomyces* Mongkols. et al., *Bhushaniella* Mongkolsamrit et al., *Hirsutella* Pat., *Hymenostilbe* Petch, *Ophiocordyceps* Petch, *Purpureocillium* Luangsa-ard et al., *Clonostachys* Corda and *Chlorocillium* Zare & W. Gams, from spiders. Crous et al. (2023) introduced a new araneogenous genus, *Gamszarella* Crous, while Khonsanit et al. (2024) introduced two new spider-host genera, *Arachnidicola* Khons. et al., and *Corniculantispora* Khons., et al. and restricted the hosts of *Akanthomyces* to moths. Thus, the araneogenous fungi were distributed in 21 genera of Hypocreales (List of the genera see Suppl. material 1) (Crous et al. 2023; Khonsanit et al. 2024; Wang et al. 2024a).

The karst region in southwestern China is one of the 36 biodiversity hotspots in the world (Delgado-Baquerizo et al. 2020). A large range of continuously distributed primary forests exist in the karst region, with an exceptionally diverse ecosystem. As a result of the complex ecological environment and unique geographic conditions in this region, unique species can be described (Özkan et al. 2010; Su et al. 2017). Seventeen new araneogenous species and new records were reported by Chen et al. (2012, 2016a, b, 2017a, b, 2018, 2019a, 2022a, b, c, 2023, 2024), Han et al. (2013) and Zou et al. (2016, 2021) from karst region of southwest China.

During a survey of araneogenous fungi and their allies in southwestern China, infected spider specimens were collected, and fungal strains were isolated and purified. Isolated strains were identified based on the multigene phylogeny and morphological characteristics, and introduced four new species in Clavicipitaceae and Cordycipitaceae i.e. *Chlorocilliumguizhouense* sp. nov., *C.vallense* sp. nov., *Gamszarellasinensis* sp. nov., and *G.vallensis* sp. nov. Moreover, *Gamszarellaantillana* is not congeneric with *Gamszarella* s. str. in the phylogenetic analyses, thus, *Neogamszarella* is proposed to accommodate it.

## ﻿Materials and methods

### ﻿Specimen collection and identification

The specimens were collected from Dali Dong Village (26°01'58.70"N, 108°24'48.06"E), Rongjiang County, Qiandongnan Miao and Dong Autonomous Prefecture, Mayao River Valley (26°21'24.71"N, 107°22'48.22"E), Duyun City, Qiannan Buyi and Miao Autonomous Prefecture and Bala Valley (26°45'7.0344"N, 106°58'57.09"E), Wudang District, Guiyang, Guizhou Province, on 1^st^ October 2018, 4^th^ September 2021 and 5^th^ April 2024, respectively. The samples were placed in an ice box and brought to the laboratory. Specimens were preserved in the refrigerator at 4 °C until further processing. The surface of each arthropod body was rinsed with sterile water, followed by sterilization with 75% ethanol for 3–5 s and rinsing again three times with sterilized water. After drying on sterilized filter paper, a piece of the synnema, mycelium or sclerotium was cut from the specimen and placed on plates of potato dextrose agar (PDA) or PDA modified by the addition of 1% w/v peptone containing 0.1 g/l streptomycin and 0.05 g/l tetracycline (Chen et al. 2019b). After fungal colonies emerged from the plated samples, a piece of mycelium from the colony edge was transferred onto new agar plates and cultured at 25 °C for 14 days under 12 h light/12 h dark conditions (Zou et al. 2010). The holotypes and ex-types cultures were deposited at the Institute of Fungus Resources, Guizhou University (formally Herbarium of Guizhou Agricultural College; code, GZAC), Guiyang City, Guizhou, China. MycoBank numbers were obtained as outlined in MycoBank (http://www.MycoBank.org) (Crous et al. 2004).

Colony characteristics were determined on PDA cultures incubated at 25 °C for 14 days and growth rate, presence of octahedral crystals and colony colors (surface and reverse) were observed. To investigate microscopic characteristics, a little of the mycelia was picked up from the colony and mounted in lactophenol cotton blue or 20% lactic acid solution and the asexual morphological characteristics (e.g., conidiophores, phialides or conidiogenous cells, and conidia) were observed and measured using a Leica DM4 B microscope.

### ﻿DNA extraction, polymerase chain reaction amplification and nucleotide sequencing

DNA extraction was carried out using a fungal genomic DNA extraction kit (DP2033, BioTeke Corporation) according to Liang et al. (2011). The extracted DNA was stored at −20 °C. Polymerase chain reaction (PCR) was used to amplify genetic markers using the following primer pairs: ITS4/ITS5 for the internal transcribed spacer (ITS) region (White et al. 1990), LR0R/LR5 for 28s large subunit ribosomal (LSU) (Vilgalys and Hester 1990), fRPB2-5F/fRPB2-7cR for RNA polymerase II second largest subunit (*RPB2*) (Liu et al. 1999) and 983F/2218R for translation elongation factor 1 alpha (*tef-1α*) (Castlebury et al. 2004). The thermal cycle of PCR amplification for these phylogenetic markers was set up following the procedure described by Chen et al. (2021). PCR products were purified and sequenced at Sangon Biotech (Shanghai) Co. All newly generated sequences were deposited in GenBank and accession numbers were obtained (Table 1).

**Table 1.** List of strains and GenBank accession numbers of sequences used in this study.

Species	Strain	GenBank Accession No.			
		ITS	LSU	*RPB2*	*tef*-1α
*Aciculosporiumoplismeni*	MAFF 246966	LC571760	LC571760	-	LC572040
*A.take*	MAFF 241224	LC571753	LC571753	-	LC572034
*A.take*	TNS-F-60465	LC571755	LC571756	-	LC572035
*Akanthomycesaculeatus*	HUA 186145 ^T^	-	MF416520	-	MF416465
*A.aculeatus*	TS 772	-	KC519370	-	KC519366
*Aschersoniaconfluens*	BCC 7961	JN049841	DQ384947	-	DQ384976
*A.placenta*	BCC 7869	JN049842	EF469074	-	EF469056
*Ascopolyporusalbus*	BCC 48975 ^T^	OL331502	OL322048	OL322065	OL322035
*A.albus*	BCC 48976	OL331503	OL322049	OL322066	OL322036
*Arachnidicolasulphurea*	TBRC 7248 ^T^	NR_164419	MF140722	MF140812	MF140843
*A.sulphurea*	TBRC 7249	MF140757	MF140721	MF140734	MF140842
*Atkinsonellahypoxylon*	B4728	-	-	-	KP689546
*Balansiaepichloe*	A.E.G. 96-15a	-	-	-	EF468743
*B.henningsiana*	A.E.G. 96-27a	JN049815	AY545727	-	AY489610
*B.pilulaeformis*	A.E.G. 94-2	-	AF543788	-	DQ522319
*Beauveriabassiana*	ARSEF 1564^T^	HQ880761	-	HQ880905	HQ880974
*B.bassiana*	ARSEF 1478	AY531981	-	HQ880908	AY531890
*Blackwellomycescardinalis*	OSC 93610	JN049843	AY184963	EF469106	EF469059
*B.cardinalis*	OSC93609^T^	NR_159788	AY184962	DQ522422	DQ522325
*Bhushaniellarubra*	BCC 47541^T^	OQ892128	OQ892133	OQ914433	OQ914428
*B.rubra*	BCC 47542	OQ892129	OQ892134	OQ914434	OQ914429
*Chlorocilliumaraneogenum*	DY101801^T^	MW730536	MW730623	-	MW753039
*C.araneogenum*	DY101802	MW730545	MW730625	-	MW753040
*C.griseum*	CBS 387.73^T^	KU382150	KU382218	-	-
*C.griseum*	RCEF6632	MW031768	MW084342	-	MW091327
*C.gueriniae*	BRIP 72680a^T^	OR750699	OR731505	-	OR737799
*C.gueriniae*	BRIP 72666a	OR750701	OR731507	-	OR737801
*C.gueriniae*	BRIP 72668a	OR750702	OR731508	-	OR737802
*C.lepidopterorum*	SD05361^T^	MW730543	MW730624	-	MW753041
*C.lepidopterorum*	SD05362	MW730611	MW730629	-	MW753042
*C.montefioreae*	BRIP 70299a^T^	PP420202	PP415875	-	PP438400
***C.guizhouense***	**DL10171^T^**	**MN128448**	-	-	**MN101596**
***C.guizhouense***	**DL10172**	**PQ432742**	-	-	**PQ444210**
*C.sinense*	KY07181^T^	PP768154	PP768156	-	PP766580
*C.sinense*	KY07182	PP768155	PP768157	-	PP766581
***C.vallense***	**DY09021^T^**	**PQ432743**	**PQ432746**	-	**PQ444211**
***C.vallense***	**DY09022**	**PQ432744**	**PQ432747**	-	**PQ444212**
*Clavicepsfusiformis*	ATCC 26019	JN049817	U17402	-	DQ522320
*C.purpurea*	GAM 12885	U57669	AF543789	-	AF543778
*C.purpurea*	S.A. cp11	-	EF469075	-	EF469058
*Clonostachysrosea*	GJS90-227	-	AY489716	-	AY489611
*Collarinaaurantiaca*	FMR 11134	KJ807178	KJ807181	-	-
*C.aurantiaca*	FMR 11784	KJ807177	KJ807180	-	-
*Conoideocrellaluteorostrata*	NHJ 11343	JN049859	EF468850	-	EF468801
*C.luteorostrata*	NHJ 12516	JN049860	EF468849	-	EF468800
*C.tenuis*	NHJ 6293	JN049862	EU369044	-	EU369029
*Corallocytostromaornithocopreoides*	WAC 8705	-	-	-	LT216546
*Cordycepsmilitaris*	OSC 93623 ^T^	JN049825	AY184966	-	DQ522332
*C.militaris*	YFCC 6587	-	MN576818	MN576932	MN576988
*C.inthanonensis*	BCC 55812 ^T^	MT000706	MT003041	MT017832	-
*C.inthanonensis*	BCC 56302	MT000705	MT003040	MT017831	MT017853
*Corniculantisporapsalliotae*	CBS 532.81 ^T^	MH861374	AF339560	EF469112	EF469067
*C.psalliotae*	CBS 101270	-	EF469081	EF469113	EF469066
*Corpulentisporamagnispora*	CGMCC 3.19304 ^T^	MK329102	MK329007	MK335985	MK336037
*C.magnispora*	LC12469	MK329103	MK329008	MK335986	MK336038
Dussiellatuberiformis*		-	-	-	JQ257027
*Engyodontiumaranearum*	CBS 309.85	JN036556	AF339526	DQ522439	DQ522341
*Ephelisjaponica*	CBS 236.64	MH858427	-	-	-
*E.japonica*	Eph.oryzae	AB038564	-	-	-
*E.tripsaci*	CBS 857.72^T^	NR_153997	NG_059240	-	-
*E.elymi*	C. Schardl 760	-	AY986924	-	AY986951
*Epichloetyphina*	ATCC 56429	JN049832	U17396	-	AF543777
*Flavocilliumacerosum*	CBS 418.81^T^	NR_111268	KM283786	KM283852	KM283810
*F.bifurcatum*	YFCC 6101^T^	MN576833	MN576781	MN576897	MN576951
*Gamszareahumicola*	CGMCC 3.19303^T^	NR_172830	NG_075268	MK335979	MK336027
*G.wallacei*	CBS 101237^T^	NR_111267	NG_042398	EF469119	EF469073
*Gamszarellabuffelskloofina*	CBS 150062^T^	OR680769	OR717025	OR683726	
***G.sinensis***	**WD04081^T^**	**PQ527895**	**PQ527899**	**PQ553218**	**PQ553220**
***G.sinensis***	**WD04082**	**PQ527896**	**PQ527900**	**PQ553219**	**PQ553221**
***G.vallensis***	**WD04101^T^**	**PQ527897**	**PQ527901**	-	-
***G.vallensis***	**WD04102**	**PQ527898**	**PQ527902**	-	-
*Gibellulanigeli*	NHJ 10808^T^	-	EU369035	EU369076	EU369018
*G.nigeli*	BCC47555	MH532885	-	-	MH521897
*Helicocollumsurathaniensis*	BCC 34463	-	KT222328	-	KT222336
*H.surathaniensis*	BCC 34464^T^	-	KT222329	-	KT222337
*Heteroepichloebambusae*	Ba-01	AB065426	-	-	-
*H.bambusae*	Bo-01	AB065428	-	-	-
*H.sasae*	*E.sasae*-H	AB065432	-	-	-
*H.sasae*	*E.sasae*-N	AB065431	-	-	-
*Hevansianovoguineensis*	CBS 610.80^T^	NR_169678	NG_070837	MH521844	MH521885
*H.novoguineensis*	BCC 47881	JX192685	MH394650	MH521845	MH521886
*Hyperdermiumbertonii*	AF242354	-	AF242354	-	-
*Jenniferiacinerea*	BCC 06839	GQ249999	GQ249970	EU369070	EU369009
*J.cinerea*	BCC 2191	GQ250000	GQ249971	-	GQ250029
*Kanoksriazaquensis*	HMAS 246915^T^	MT789699	MT789697	-	MT797812
*K.zaquensis*	HMAS 246917	MT789698	MT789696	-	MT797811
*Keithomycescarneus*	CBS 239.32	NR_131993	NG_057769	-	EF468789
*Lecanicilliumlecanii*	CBS 101247	JN049836	AF339555	DQ522466	DQ522359
*L.lecanii*	CBS 102067^T^	MH862778	KM283795	KM283860	KM283818
*Leptobacilliumchinense*	CGMCC 3.14970^T^	NR_155782	NG_069101	-	-
*L.coffeanum*	COAD 2057^T^	MF066035	MF066033	-	-
*L.coffeanum*	COAD 2061	MF066034	MF066032	-	-
*L.filiforme*	URM 7918^T^	NR_171744	NG_075252	-	-
*L.leptobactrum*	CBS 774.69^T^	NR_154111	NG_069745	-	-
*Liangiasinensis*	YFCC 3103^T^	MN576831	MN576782	MN576898	MN576952
*L.sinensis*	YFCC 3104	MN576832	MN576783	MN576899	MN576953
*Marquandomycesmarquandii*	CBS 182.27	NR_131994	EF468845	-	EF468793
*Metapochoniabulbillosa*	CBS 145.70	MH859529	AF339542	-	EF468796
*M.gonioides*	CBS 891.72	AJ292409	AF339550	-	DQ522354
*M.rubescens*	CBS 464.88^T^	-	AF339566	-	EF468797
*M.sulchlasporia*	CBS 251.83	NR_154139	MH873311	-	KJ398790
*Metarhiziopsismicrospora*	CEHS133a	EF464589	EF464571	-	-
*M.microspora*	INEHS133a	EF464583	EF464572	-	-
*Metarhiziumanisopliae*	ARSEF 7487	HQ331446	-	-	DQ463996
*M.anisopliae*	CBS 130.71^T^	MT078884	MT078853	-	MT078845
*M.flavoviride*	CBS 125.65	MT078885	MT078854	-	MT078846
*M.flavoviride*	CBS 700.74	-	MT078855	-	MT078847
*M.flavoviride*	CBS 218.56^T^	MH857590	MH869139	-	KJ398787
*Microhilumoncoperae*	ARSEF 4358	-	AF339532	EF468936	EF468785
*Moelleriellaphyllogena*	CUP 067785	-	EU392610	-	EU392674
*M.phyllogena*	CUP 067793	-	EU392608	-	EU392672
*M.umbospora*	CUP 067817^T^	-	EU392628	-	EU392688
*Morakotiafusca*	BCC 64125	-	KY794862	-	KY794857
*M.fusca*	BCC 79272^T^	-	KY794861	-	KY794856
*Mycophilomycespericoniae*	CPC 27558	NR_154209	NG_059746	-	-
*Myriogenosporaatramentosa*	A.E.G 96-32	-	AY489733	-	AY489628
*Neoaraneomycesaraneicola*	DY101711^T^	MW730520	MW730609	-	MW753033
*N.araneicola*	DY101712	MW730522	MW730610	-	MW753034
*Neobaryopsisandensis*	A.F.25967-2^T^	NR_169713	NG_068701	-	-
*N.andensis*	A.F.25967-1	MT153956	MT153985	-	-
*Neobaryaparasitica*	Marson s/n	KP899626	KP899626	-	-
*Neogamszarellaantillata*	CBS 350.85^T^	NR_111097	AF339536	DQ522450	DQ522350
*Neohyperdermiumpiperis*	CBS 116719^T^	-	AY466442	EU369083	DQ118749
*N.pulvinatum*	P.C. 602	-	DQ118738	AF242353	DQ118746
*Neotorrubiellachinghridicola*	BCC 80733^T^	NR_175629	MK632097	MK632149	-
*N.chinghridicola*	BCC 39684	MK632038	MK632096	-	MK632072
*Niessliaexilis*	CBS 560.74	-	AY489720	-	AY489614
*Nigeliaaurantiaca*	BCC13019	-	GU979948	-	GU979957
*N.martiale*	EFCC 6863	-	JF415974	-	JF416016
*Niveomycescoronatus*	NY04434800^T^	-	ON493606	ON513400	ON513397
*N.coronatus*	Niveo	-	ON493605	-	-
*Orbiocrellapetchii*	NHJ 6209	JN049861	EU369039	-	EU369023
*O.petchii*	NHJ 6240	-	EU369038	-	EU369022
*Papiliomycesliangshanensis*	EFCC 1452	-	EF468815	-	EF468756
*P.liangshanensis*	EFCC 1523	-	EF468814	-	EF468755
*P.shibinensis*	GZUH SB13050311^T^	NR154178	-	-	KR153589
*Parahevansiakoratensis*	BCC01485	GQ250010	GQ249981	-	GQ250031
*P.websteri*	NHJ 2662	GQ250008	GQ249982	-	GQ250032
*Parametarhiziumchangbaiense*	CGMCC 19143^T^	MN589741	MN589994	-	MN908589
*P.hingganense*	CGMCC 19144	MN055703	MN061635	-	MN065770
*Paraneoaraneomycessinensis*	ZY 22.006	OQ709254	OQ709260	-	OQ719626
*P.sinensis*	ZY 22.007	OQ709255	OQ709261	-	OQ719627
*Parengyodontiumalbum*	CBS 836.71	LC092882	MH872118	-	LC382178
*P.album*	CBS 368.72	MH860502	MH872217	-	LC382183
*Parepichloecinerea*	Ne-01	AB065425	-	-	-
*Periglandulaipomoeae*	IasaF13	-	-	-	KP689568
*Pleurodesmosporacoccorum*	CBS 460.73	MH860743	MH872455	-	-
*P.lepidopterorum*	DY 10501^T^	MW826576	-	MW834316	MW834317
*P.lepidopterorum*	DY 10502	MW826577	-	MW834318	MW834319
*Pochoniaboninensis*	JCM 18597	AB709858	AB709831	-	AB758463
*P.chlamydosporia*	CBS 101244	JN049821	DQ518758	-	DQ522327
*Polystromomycesaraneae*	BCC 93301^T^	MZ684101	MZ684016	MZ707845	MZ707825
*Pseudogibellulaformicarum*	BCC 84257	MT508782	MT512653	-	MT533480
*P.formicarum*	CBS 433.73	MH860731	MH872442	-	MT533481
*Pseudoniveomycesarachnovorum*	BCC 95818^T^	OR098526	-	-	OR133173
*P.blattae*	BCC 53567^T^	ON103042	ON103167	ON125036	-
*P.blattae*	BCC 53568	ON103043	ON103168	ON125037	ON125025
*Pseudolecanicilliumcaatingaense*	URM8446^T^	ON862933	ON862925	OP290514	OP290526
*P.caatingaense*	URM8442	ON862934	ON862926	OP290513	OP290525
*Purpureomycesmaesotensis*	BCC 88441	MN781916	MN781877	-	MN781734
*P.maesotensis*	BCC 85349	MN781928	MN781872	-	MN781729
*P.maesotensis*	BCC 89300^T^	MN781917	MN781876	-	MN781733
*Regiocrellacamerunensis*	ARSEF 7682	-	DQ118735	-	DQ118743
*Rotiferophthoraangustispora*	CBS 101437	AJ292412	AF339535	-	AF543776
*Samsoniellaalboaurantium*	CBS 262.58^T^	AY624179	AB080087	MF416448	MF416497
*S.alboaurantium*	CBS 240.32	AY624178	JF415979	JF415999	JF416019
*S.inthanonensis*	TBRC 7915^T^	NR_164420	NG_069500	MF140815	MF140849
*S.inthanonensis*	TBRC 7916	MF140760	MF140724	MF140814	MF140848
*Samuelsiachalalensis*	CUP 067856^T^	-	EU392637	-	EU392691
*S.mundiveteris*	BCC 40021	-	GU552152	-	GU552145
*S.rufobrunnea*	CUP 067858	-	AY986918	-	AY986944
*Shimizuomycesparadoxus*	EFCC 6279	JN049847	EF469084	-	EF469071
*S.paradoxus*	EFCC 6564	-	EF469083	-	EF469072
*Simplicilliumlanosoniveum*	CBS 123.42^T^	NR_171734	NG_068571	-	-
*Sungiayongmunensis*	EFCC 2131	JN049856	EF468833	-	EF468770
*S.yongmunensis*	EFCC 2135	-	EF468834	-	EF468769
*Tyrannicordycepsfratricida*	TNS-F 19011	JQ349068	JQ257023	-	JQ257028
*Ustilaginoideadichromonae*	MRL IB9228	-	-	-	JQ257025
*U.virens*	ATCC 16180	-	-	-	JQ257026
*U.virens*	MAFF 240421	-	JQ257011	-	JQ257024
*Yosiokobayasiakusanagiensis*	TNS-F18494	-	JF415972	-	JF416014
*Zareafungicola*	CBS 992.69^T^	NR_119653	KM283792	KM283857	KM283816
*Zouiacauligalbarum*	GZUIFRZHJ01^T^	MH730663	MH730667	MH801924	MH801920
*Z.cauligalbarum*	GZUIFRZHJ02	MH730664	MH730668	MH801925	MH801921
*Pleurocordycepsaurantiaca*	MFLUCC 17-2113	MG136916	MG136910	-	MG136875
*P.marginaliradians*	MFLU 17-1582^T^	MG136920	MG136914	-	MG136878
*Purpureocilliumlilacinum*	CBS 431.87	AY624188	EF468844	EF468940	EF468791

Note: New strains or species are in bold type. * J.F. White, Scale on Arundinaria tecta, North Carolina, 2000. “^T^” denotes ex-type. Abbreviations: ARSEF, USDA-ARS Collection of Entomopathogenic Fungal cultures, Ithaca, NY; ATCC, American Type Culture Collection, USA; BCC, BIOTEC Culture Collection, KlongLuang, Thailand; CBS, Centraalbureau voor Schimmelcultures, Utrecht, the Netherlands; CGMCC, China General Microbiological Culture Collection Center, China; EFCC, Entomopathogenic Fungal Culture Collection, Chuncheon, Korea; GZUH, Guizhou University Herbarium, Guiyang, Guizhou, China; HMAS, Herbarium of Mycology, Chinese Academy of Sciences; MAFF, Ministry of Agriculture, Forestry and Fisheries of Japan, Tokyo, Japan; NHJ, Nigel Hywel-Jones personal collection; OSC, Oregon State University Herbarium, Corvallis; TNS-F, the mycological herbarium of the National Museum of Nature and Science, Tsukuba, Ibaraki, Japan; WAC, Western Australian Plant Pathology Reference Culture Collection, Australia, Perth.

### ﻿Sequence alignments and phylogenetic analyses

DNASTAR™ Lasergene (v 6.0) was used to edit DNA sequences in this study. Analyses 1: ITS, LSU and *tef*-1α sequences for the strains in Clavicipitaceae were downloaded from GenBank based on Xiao et al. (2023), Chen et al. (2024) and other sequences were selected based on BLASTn searches. Analyses 2: ITS, LSU, *RPB2* and *tef*-1α sequences for the strains in Cordycipitaceae were downloaded from GenBank based on Khonsanit et al. (2024) and other sequences were selected based on BLASTn searches. All the sequences were aligned and edited by MAFFT v.7.037b (Katoh and Standley 2013) and MEGA6 (Tamura et al. 2013). Combined sequences for analysis 1 (dataset 1: ITS, LSU, *tef*-1α) and analysis 2 (dataset 2: ITS, LSU, *RPB2*, *tef*-1α) were obtained using SequenceMatrix v.1.7.8 (Vaidya et al. 2011). The model was selected for Bayesian analysis by ModelFinder (Kalyaanamoorthy et al. 2017) in PhyloSuite (v1.2.2) software (Zhang et al. 2020).

The datasets 1 and 2 for analysis 1 and 2 were analyzed using Bayesian inference (BI) and maximum likelihood (ML) methods, respectively. For BI, a Markov chain Monte Carlo (MCMC) algorithm was used to generate phylogenetic trees with Bayesian probabilities for the combined sequence datasets using MrBayes v.3.2 (Ronquist et al. 2012). The Bayesian analysis resulted in 20,001 trees after 10,000,000 generations. The first 4,000 trees, representing the burn-in phase of the analysis, were discarded, while the remaining 16,001 trees were used to calculate posterior probabilities in the majority rule consensus tree. After the analysis was finished, each run was examined if it was greater than 200 using the program Tracer v.1.5 (Drummond and Rambaut 2007) to determine burn-in and confirm that both runs had converged. ML analyses were constructed with IQ-TREE (v 2.0) (Trifinopoulos et al. 2016), using an automatic selection of the model according to BIC.

### ﻿Genealogical Concordance Phylogenetic Species Recognition (GCPSR) analysis

The Genealogical Concordance Phylogenetic Species Recognition model was applied to analysis the related species. The pairwise homoplasy index (PHI) (Bruen et al. 2006) is a model test based on the fact that multiple gene phylogenies will be concordant between species and discordant due to recombination and mutations within a species. The test was performed in SplitsTree4 (Huson and Bryant 2006) as described by Quaedvlieg et al. (2014) to determine the recombination level within phylogenetically closely-related species using a three-locus or four-locus concatenated dataset. The new species and their closely-related species were analyzed using this model. The relationships between closely-related species were visualized by constructing a split graph, using both the LogDet transformation and splits decomposition options.

## ﻿Results

### ﻿Phylogenetic analyses

**Figure 1.**
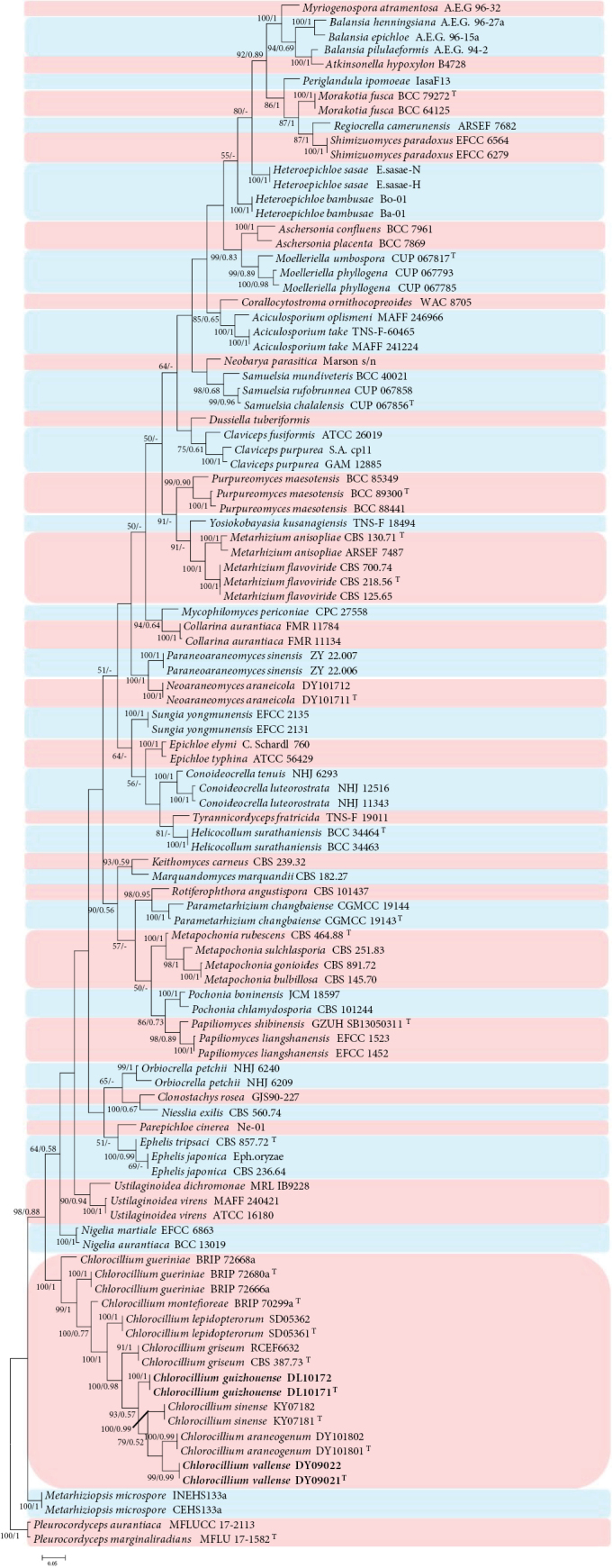
Phylogram retrieved from IQTREE of the new species and related species in Clavicipitaceae using the combined dataset of ITS, LSU, and *tef*-1α gene regions. The statistical values are provided at nodes as ML/PP (ML value above 50% and BI value above 0.50). The tree is rooted with *Pleurocordycepsaurantiaca* (MFLUCC 17-2113) and *P.marginaliradians* (MFLU 17-1582). Ex-types, new strains and new species are indicated by the superscript “T” and in bold, respectively.

Analysis 1: Phylogenetic trees were generated in analysis 1 to determine the establishment of the new *Chlorocillium* species in Clavicipitaceae (Fig. 1). *Pleurocordycepsaurantiaca* (Y.P. Xiao et al.) Y.H. Wang et al. (MFLUCC 17-2113) and *P.marginaliradians* (Y.P. Xiao et al.) Y.H. Wang et al. (MFLU 17-1582) were used as the outgroup taxa in the analysis. The dataset included 74 taxa, and consisted of 2,280 (ITS, 660; LSU, 740, and *tef*-1α, 880) characters with gaps.

The selected model for ML analysis was TIM+F+I+G4. The final value of the highest scoring tree was –26,825.060, which was obtained from the ML analysis of the dataset. The parameters of the GTR model used to analyze the dataset were estimated based on the following frequencies: A = 0.224, C = 0.291, G = 0.279, T = 0.207; substitution rates AC = 1.00000, AG = 2.11049, AT = 1.16800, CG = 1.16800, CT = 5.30232 and GT = 1.00000, as well as the gamma distribution shape parameter α = 0.474. The selected model of the dataset for BI analysis was GTR+F+I+G4. The phylogenetic tree (Fig. 1) constructed using ML and BI analyses was largely congruent and strongly supported in most branches. Most genera clustered into independent clades. Strains DL10171, DL10172, DY09021 and DY09022 clustered into two independent clades. Strains DL10171 and DL10172 have a close relationship with *Chlorocilliumgriseum*, whereas strains DY09021 and DY09022 have a close relationship with *C.sinense* W.H. Chen et al. and *C.araneogenum* (W.H. Chen et al.) W.H. Chen, et al.

**Figure 2.**
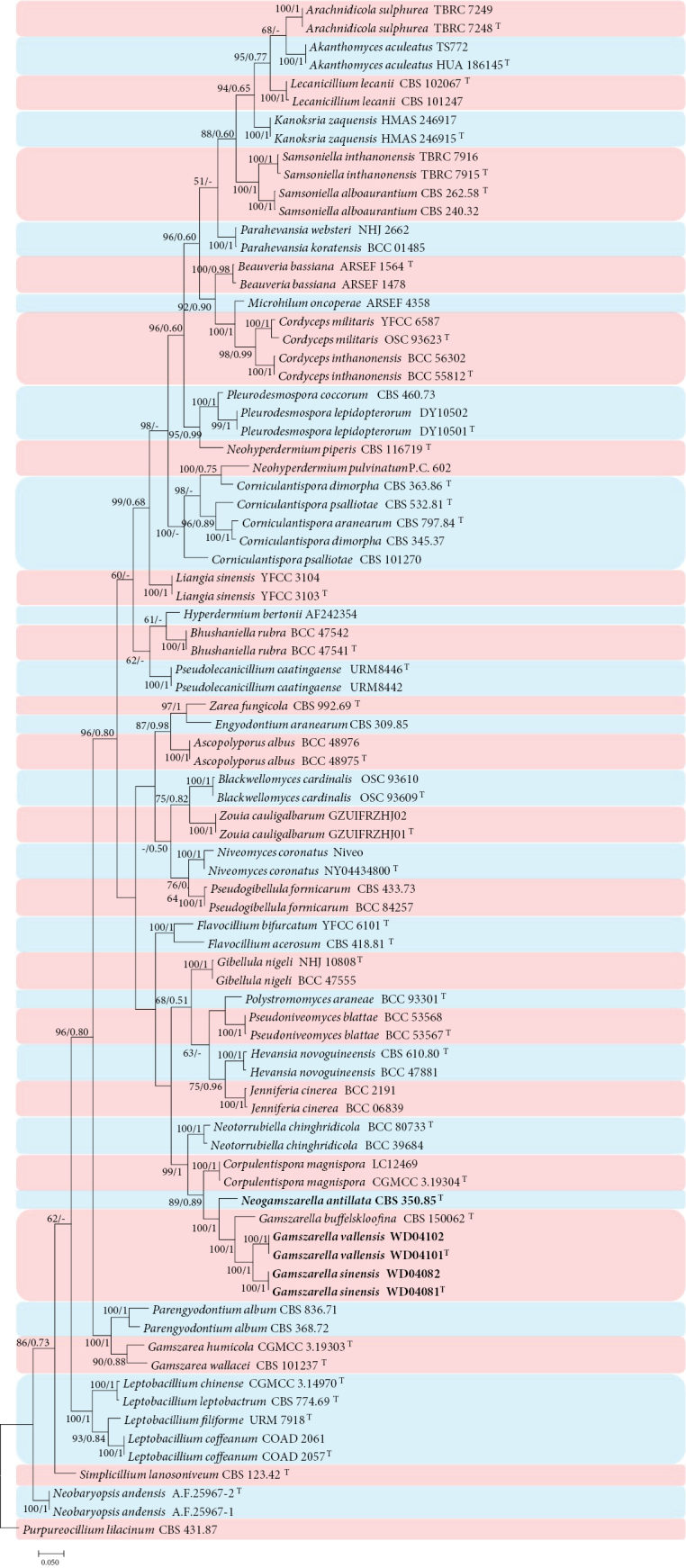
Phylogram retrieved from IQTREE of the new species and other related species in Cordycipitaceae using the combined dataset of ITS, LSU, *RPB2* and *tef*-1α gene regions. The statistical values are provided at nodes as ML/PP (ML value above 50% and BI value above 0.50). The tree is rooted with *Purpureocilliumlilacinum* (CBS 431.87). Ex-types, new strains and new species are indicated by the superscript “T” and in bold, respectively.

Analysis 2: The phylogenetic trees were generated in analysis 2 to determine the establishment of the new species in Cordycipitaceae (Fig. 2). *Purpureocilliumlilacinum* (Thom) Luangsa-ard et al. (CBS 431.87) was used as the outgroup taxon in the analysis. The dataset included 49 taxa and consisted of 2,280 (ITS, 631; LSU, 782, *RPB2*, 833 and *tef*-1α, 893) characters with gaps.

The selected model for ML analysis was TIM3+F+I+G4. The final value of the highest scoring tree was –36,309.393, which was obtained from the ML analysis of the dataset. The parameters of the GTR model used to analyze the dataset were estimated based on the following frequencies: A = 0.237, C = 0.280, G = 0.272, T = 0.211; substitution rates AC = 1.16611, AG = 2.87992, AT = 1.00000, CG = 1.16611, CT = 6.74648 and GT = 1.00000, as well as the gamma distribution shape parameter α = 0.445. The selected model for BI analysis was GTR+F+I+G4 (ITS), GTR+F+I+G4 (LSU and *tef*-1α) and SYM+G4 (*RPB2*). The phylogenetic trees (Fig. 2) constructed using ML and BI analyses were largely congruent and strongly supported in most branches. Most genera clustered into independent clades. Strains WD04081, WD04082, WD04101 and WD04102 clustered into two independent clades with high statistical support (100% ML/1 PP) and were clustered with *Gamszarellaantillana* (R.F. Castañeda & G.R.W. Arnold) Crous and *G.buffelskloofina* Crous in a clade with high statistical support in ML and BI analysis (100% ML/1 PP).

### ﻿Genealogical Concordance Phylogenetic Species Recognition (GCPSR) analysis

**Figure 3.**
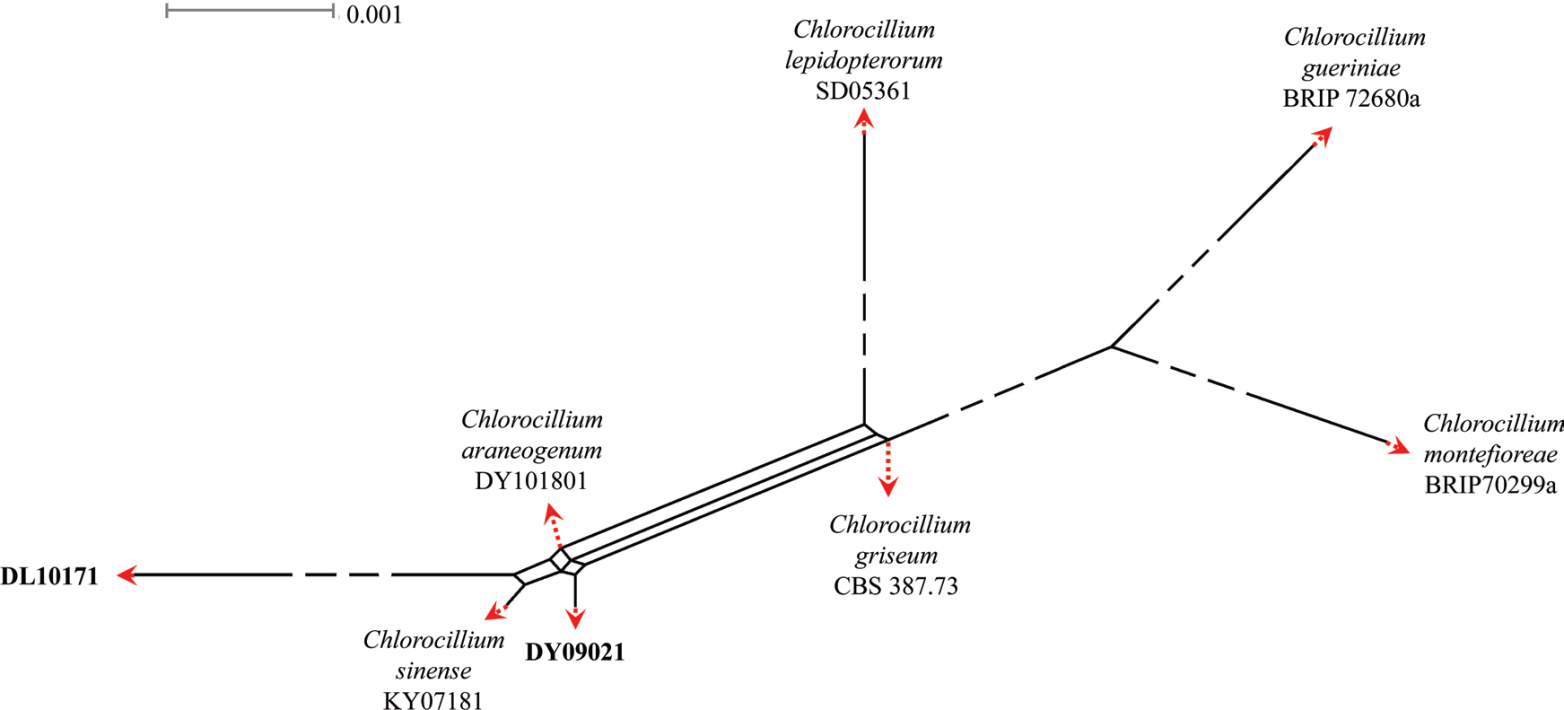
Results of the pairwise homoplasy index (PHI) test of the new *Chlorocillium* strains and its closely-related species using both LogDet transformation and splits decomposition. PHI test results (Φ_w_) < 0.05 indicate significant recombination within the dataset. The new strains are in bold type.

**Figure 4.**
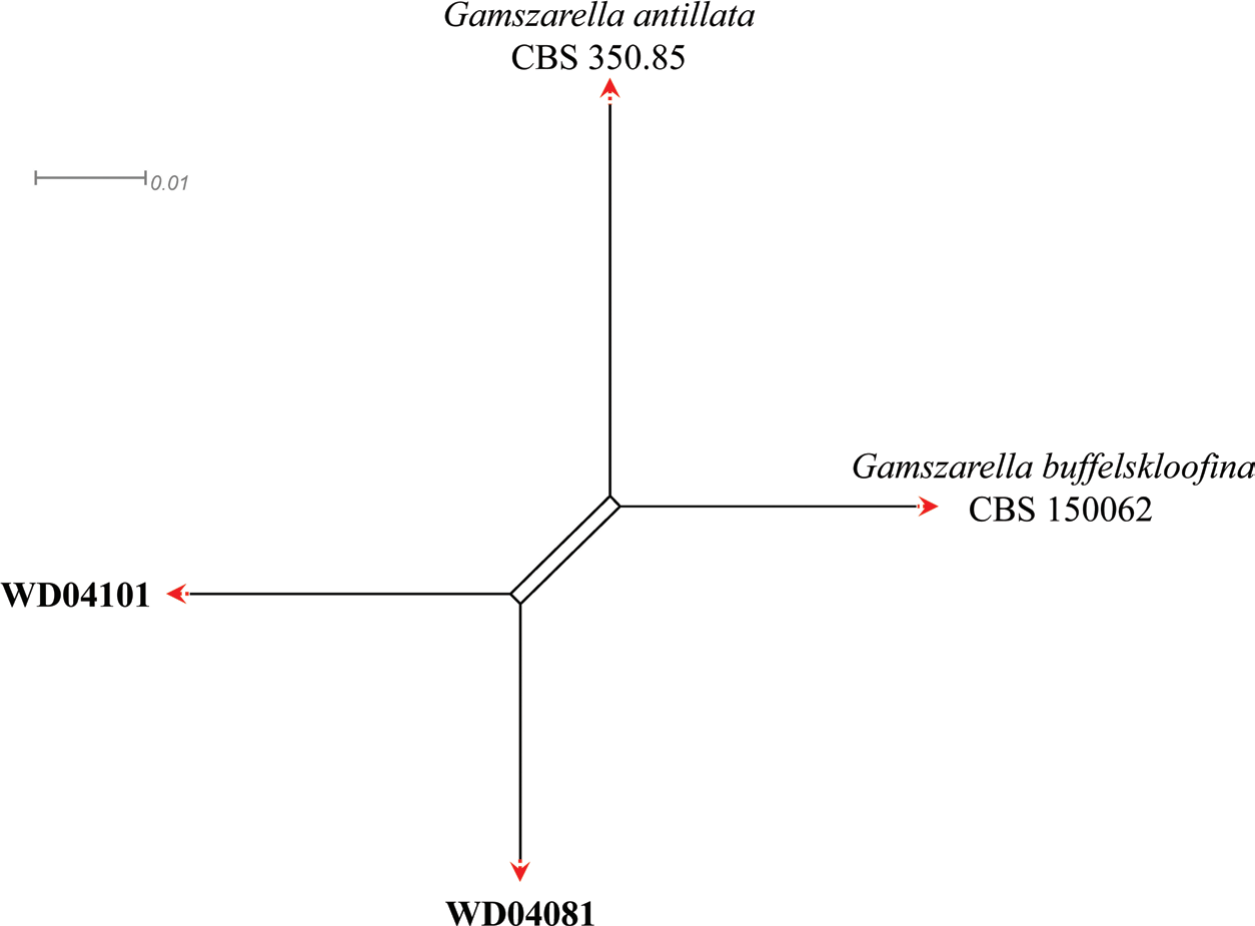
Results of the pairwise homoplasy index (PHI) test of the new *Gamszarella* strains and its closely-related species using both LogDet transformation and splits decomposition. PHI test results (Φ_w_) < 0.05 indicate significant recombination within the dataset. The new strains are in bold type.

A three-locus concatenated dataset (ITS, LSU and *tef*-1α) was used to determine the recombination level within *Chlorocilliumaraneogenum* (DY101801), *C.griseum* (CBS 387.73), *C.gueriniae* (BRIP 72680a), *C.montefioreae* (BRIP70299a), *C.lepidopterorum* (SD05361), *C.guizhouense* (DL10171), and strains KY07181 and DY09021 (Fig. 3), whereas a four-locus concatenated dataset (ITS, LSU, *RPB2* and *tef*-1α) was used to determine the recombination level within *Gamszarellaantillana* (CBS 350.85), *G.buffelskloofina* (CBS 150062), and strains WD04081 and WD04101 (Fig. 4). Chaiwan et al. (2022) noted that if the PHI is below the 0.05 threshold (Φ_w_ < 0.05), it indicates that there is significant recombination in the dataset. This means that related species in a group and recombination levels are not different. If the PHI is above the 0.05 threshold (Φ_w_ > 0.05), it indicates that it is not significant, which means that the related species in a group level are different. The result of the pairwise homoplasy index (PHI) test of *Chlorocilliumaraneogenum*, *C.griseum*, *C.gueriniae*, *C.montefioreae*, *C.lepidopterorum*, *C.guizhouense*, strains KY07181 and DY09021 was 1.0 and revealed that those species and strains KY07181 and DY09021 were different (Fig. 3). The result of the pairwise homoplasy index (PHI) test of *Gamszarellaantillana*, *G.buffelskloofina*, and strains WD04081 and WD04101 was 1.0 and revealed that those species and strains WD04081 and WD04101 were different (Fig. 4).

### ﻿Taxonomy

#### ﻿*Chlorocillium* Zare & W. Gams, Mycol. Progr. 15: 1005, 2016

##### ﻿ Chlorocillium guizhouense

Taxon classificationFungiHypocrealesClavicipitaceae

W.H. Chen, Y.F. Han & J.D. Liang sp. nov.

D7125E89-1B5B-5137-BDA5-C25C4FEB686F

856175

Fig. 5

###### Etymology.

Referring to the location, Guizhou Province, where the type specimen was collected.

###### Type.

China • Guizhou Province, Qiandongnan Miao and Dong Autonomous Prefecture, Rongjiang County, Dali Dong Village (26°01'58.70"N, 108°24'48.06"E). On a dead spider (Araneae), 1 October 2018, Wanhao Chen, GZAC DL1017 (holotype), ex-type, DL10171.

###### Description.

Colonies on PDA reaching 21–24 mm diam. in 14 d at 25 °C, white, consisting of a basal felt and cottony, floccose overgrowth, reverse yellowish. Prostrate hyphae smooth, septate, hyaline, 1.1–1.6 μm diam. Conidial structures consisting of erect conidiophores usually arising from the aerial hyphae, solitary or lecanicillium-like with phialides in whorls of two to three. Phialides 11.6–25.3 × 1.0–1.2 μm, with a cylindrical basal portion, tapering into a distinct neck. Conidia in chains, hyaline, fusiform, 1-celled, 2.6–3.8 × 1.1–1.6 μm (average values 3.2 × 1.3 μm). Octahedral crystals not observed.

**Figure 5.**
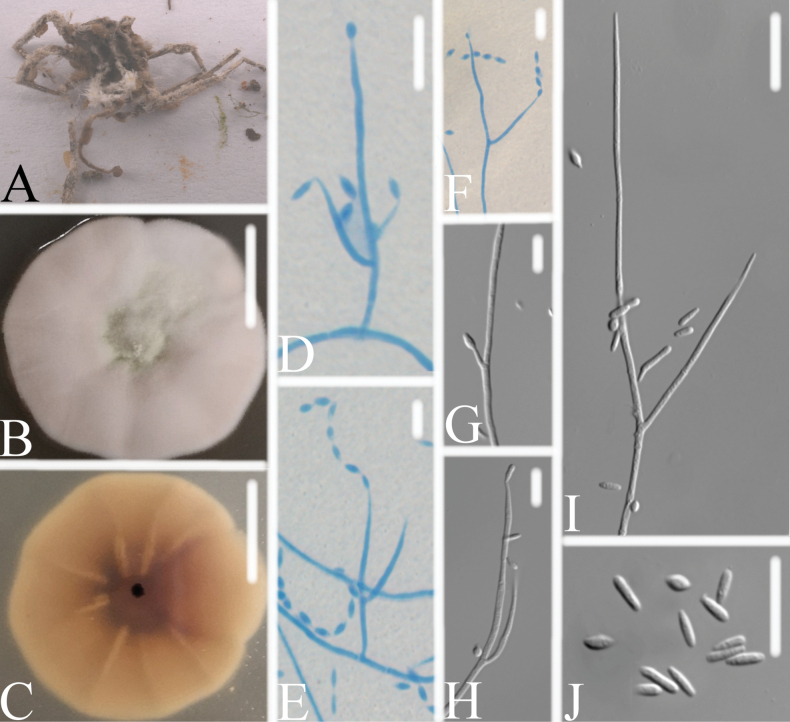
*Chlorocilliumguizhouense***A** infected spider **B, C**PDA culture plate showing top (**B**) and reverse (**C**) sides of the colony **D–F** phialides and conidia were stained with cotton blue **G**–**J** phialides and conidia. Scale bars: 10 mm (**B, C**); 10 μm (**D–J**).

###### Host.

Spider (Araneae).

###### Additional material examined.

China • Guizhou, Qiandongnan Miao and Dong Autonomous Prefecture, Rongjiang County, Dali Dong Village (26°01'58.70"N, 108°24'48.06"E). On a dead spider (Araneae), 1 October 2018, Wanhao Chen, DL10172 (living culture).

###### Remarks.

*Chlorocilliumguizhouense* was identified as *Chlorocillium*, based on the BLASTn result in NCBI and the phylogenetic analysis of the combined dataset 1 (Fig. 1). It clustered into an independent clade with a close relationship with *C.araneogenum*, *C.sinense and C.vallense* with high bootstrap value (93% ML). Compared with the typical characteristics of the known species (Table 2), *C.guizhouense* was distinguished from *C.araneogenum* by its smaller conidia (2.6–3.8 × 1.1–1.6 μm vs. 3.4–5.8 × 1.4–1.8 μm). *Chlorocilliumguizhouense* can be distinguished from *C.sinense* by its larger conidia (fusiform, 2.6–3.8 × 1.1–1.6 μm vs. fusiform to ellipsoidal, 1.9–2.9 × 0.8–1.2 μm). *Chlorocilliumguizhouense* was distinguished from *C.vallense* by its larger phialides (11.6–25.3 × 1.0–1.2 μm vs. 9.2–17.1 × 0.8–1.2 μm). Thus, the morphological characteristics and molecular phylogenetic results support *C.guizhouense* as a new species.

**Table 2.** Morphological comparison of the new species with other *Chlorocillium* species.

Species	Phialides (μm)	Conidia (μm)	Hosts	Octahedral crystals	References
*C.araneogenum*	8.3–23.3 × 1.3–2.2	fusiform, 3.4–5.8 × 1.4–1.8	spider	Absent	Chen et al. 2022a
*C.griseum*	18–40(−55) × 2–2.5	fusiform, 4.5–6 × 1.0–1.5	spider	Present	Zare and Gams 2016
*C.lepidopterorum*	21.2–33.7 × 1.1–1.4	fusiform, 3.1–4.3 × 1.3–1.5	pupa	Absent	Chen et al. 2022a
*C.sinense*	11.7–20.1 × 1.1–1.3	fusiform to ellipsoidal, 1.9–2.9 × 0.8–1.2	spider	Absent	Chen et al. 2024
***C.guizhouense***	**11.6–25.3 × 1.0–1.2**	**fusiform, 2.6–3.8 × 1.1–1.6**	**spider**	**Absent**	**This study**
***C.vallense***	**9.2–17.1 × 0.8–1.2**	**fusiform, 2.0–3.2 × 0.9–1.4**	**spider**	**Absent**	**This study**

##### ﻿ Chlorocillium vallense

Taxon classificationFungiHypocrealesClavicipitaceae

W.H. Chen, Y.F. Han & J.D. Liang sp. nov.

1CFA2FC3-1B6A-5958-B7A1-F49929920D3B

856176

Fig. 6

###### Etymology.

Referring to its location, Mayao River Valley, where the fungus was first discovered.

###### Type.

China • Guizhou Province, Qiannan Buyi and Miao Autonomous Prefecture, Duyun City, Mayao River Valley (26°21'24.71"N, 107°22'48.22"E). On a dead spider (Araneae), 4 September 2021, Wanhao Chen, GZAC DY0902 (holotype), ex-type, DY09021.

###### Description.

Colonies on PDA reaching 72–74 mm diam. in 14 days at 25 °C, white, consisting of a basal felt and cottony, floccose overgrowth, reverse yellowish. Prostrate hyphae smooth, septate, hyaline, 0.9–1.3 μm diam. Conidial structures consisting of erect branching conidiophores usually arising from the aerial hyphae, solitary or lecanicillium-like in whorls of two to four. Phialides 9.2–17.1 × 0.8–1.2 μm, with a cylindrical basal portion, tapering into a distinct neck. Conidia hyaline, fusiform, 1-celled, 2.0–3.2 × 0.9–1.4 μm (average values 2.4 × 1.2 μm). Octahedral crystals not observed.

**Figure 6.**
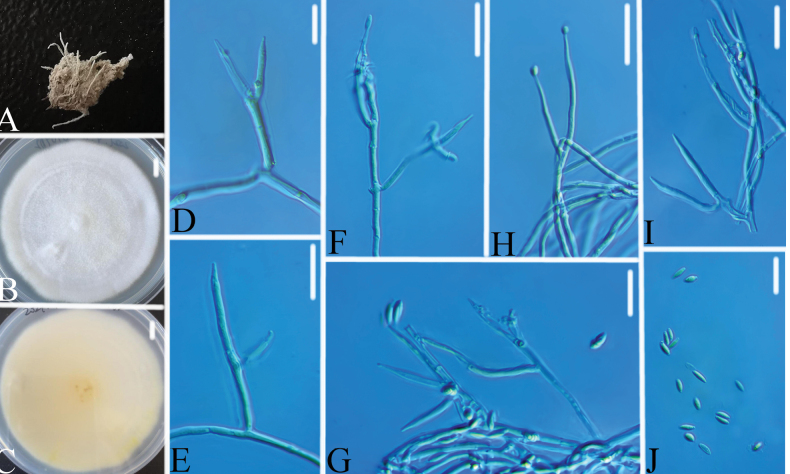
*Chlorocilliumvallense***A** infected spider **B, C**PDA culture plate showing top (**B**) and reverse (**C**) sides of the colony **D–J** phialides and conidia. Scale bars: 10 mm (**B, C**); 10 μm (**D–J**).

###### Host.

Spider (Araneae).

###### Additional strain examined.

China • Guizhou Province, Qiannan Buyi and Miao Autonomous Prefecture, Duyun City, Mayao River Valley (26°21'24.71"N, 107°22'48.22"E). On a dead spider (Araneae), 4 September 2021, Wanhao Chen, DY09022 (living culture).

###### Remarks.

*Chlorocilliumvallense* was identified as in *Chlorocilliums. str.*, based on the BLASTn result in NCBI and the phylogenetic analysis of the combined dataset 1 (Fig. 1) and clustered into an independent clade with a close relationship with *C.sinense* and *C.araneogenum*. Compared with the typical characteristics of the known species (Table 2), *C.vallense* was distinguished from *C.sinense* by its shorter phialide (9.2–17.1 × 0.8–1.2 μm vs. 11.7–20.1 × 1.1–1.3 μm), larger fusiform conidia (2.0–3.2 × 0.9–1.4 μm vs. 1.9–2.9 × 0.8–1.2 μm) and fast-growing in PDA. *Chlorocilliumvallense* was distinguished from *C.araneogenum* by its shorter phialide (9.2–17.1 × 0.8–1.2 μm vs. 8.3–23.3 × 1.3–2.2 μm) and smaller conidia (2.0–3.2 × 0.9–1.4 μm vs. 3.4–5.8 × 1.4–1.8 μm). Thus, based on both morphological characteristics and molecular phylogenetic results, we confirm *C.vallense* as a new species.

#### ﻿*Gamszarella* Crous, Persoonia 51: 391, 2023

##### ﻿ Gamszarella sinensis

Taxon classificationFungiHypocrealesClavicipitaceae

W.H. Chen, Y.F. Han & J.D. Liang sp. nov.

58A287C6-F8FD-5F6B-B019-03E60EBF424A

856388

Fig. 7

###### Etymology.

Referring to the country, China, where the fungus was first discovered.

###### Type.

China • Guizhou Province, Guiyang City, Wudang District, Bala Valley (26°45'7.0344"N, 106°58'57.09"E). On a dead spider (Araneae), 5 April 2024, Wanhao Chen, GZAC WD0408 (holotype), ex-type, WD04081.

###### Description.

Colonies on PDA reaching 37–38 mm diam in 14 d at 25 °C, white, consisting of a basal felt and cottony, floccose overgrowth, reverse yellowish, with radial patterns. Prostrate hyphae smooth, septate, hyaline, 1.5–1.9 μm diam. Conidial structures consisting of erect conidiophores usually arising from the aerial hyphae, solitary or lecanicillium-like with conidiogenous cells in whorls of two to three. Conidiogenous cells 7.0–12.0 × 1.0–1.5 μm, with a cylindrical basal portion, bearing numerous denticles, tapering into a distinct neck. Conidia hyaline, ellipsoidal to fusiform, 1-celled, 2.4–3.9 × 1.5–2.8 μm. Octahedral crystals not observed.

**Figure 7.**
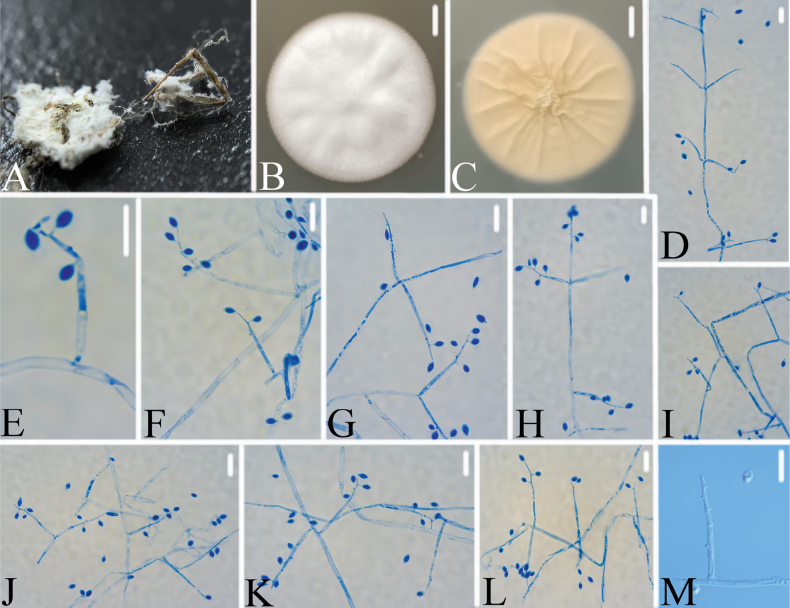
*Gamszarellasinensis***A** infected spider (Araneae) **B, C**PDA culture plate showing top (**B**) and reverse (**C**) sides of the colony **D–L** conidiogenous cells and conidia were stained with cotton blue **M** conidiogenous cells and conidia. Scale bars: 10 mm (**B, C**); 10 μm (**D–M**).

###### Host.

Spider (Araneae).

###### Additional material examined.

China • Guizhou Province, Guiyang City, Wudang District, Bala Valley (26°45'7.0344"N, 106°58'57.09"E). On a dead spider (Araneae), 5 April 2024, Wanhao Chen, WD04082 (living culture).

###### Remarks.

The new strains were identified as a member in *Gamszarella*, based on the BLASTn result in NCBI. The phylogenetic analyses of the combined dataset 2 (Fig. 2) showed that the new strains clustered as an independent clade with a close relationship to *Gamszarellaantillana* (R.F. Castañeda & G.R.W. Arnold) Crous and *G.buffelskloofina* Crous. Compared with the typical characteristics of the known species (Table 3), the new taxon, *Gamszarellasinensis* was distinguished from *G.antillana* by its shorter conidiogenous cells bearing numerous denticles (Numerous denticles, 7.0–12.0 × 1.0–1.5 μm vs. absent of denticles, 18–31 × 1 μm), ellipsoidal to fusiform conidia and absent of octahedral crystals. *Gamszarellasinensis* was distinguished from *G.buffelskloofina* by its shorter conidiogenous cells (7.0–12.0 × 1.0–1.5 μm vs. 7–22 × 1.5–2 μm), smaller ellipsoidal to fusiform conidia [2.4–3.9 × 1.5–2.8 μm vs. (3–)4–6(–10) × 2 μm] and spider host. *Gamszarellasinensis* was distinguished from *G.vallensis* by its longer conidiogenous cells (7.0–12.0 × 1.0–1.5 μm vs. 3.8–5.4 × 1.3–1.9 μm). Thus, the morphological characteristics and molecular phylogenetic results support *G.sinensis* as a new species.

**Table 3.** Morphological comparison of the new species with other *Gamszarella* species.

Species	Conidiogenous cells (μm)	Conidia (μm)	Octahedral crystals	Hosts/ substrate	References
*G.antillana*	No denticles, 18–31 × 1	Two types: primary conidia fusiform, 11–18 × 0.8–1.5; secondary conidia ellipsoidal, 3–4 × 0.8–1.2	Present	Agaric	Zare and Gams 2001
*G.buffelskloofina*	Terminating in a cluster of denticles, 7–22 × 1.5–2	Subcylindrical, (3–)4–6(–10) × 2	Absent	Insect	Crous et al. 2023
***G.sinensis***	**Numerous denticles, 7.0–12.0 × 1.0–1.5**	**Ellipsoidal to fusiform, 2.4–3.9 × 1.5–2.8**	**Absent**	**Spider**	**This study**
***G.vallensis***	**Numerous denticles, 3.8–5.4 × 1.3–1.9**	**Ellipsoidal to fusiform, 2.3–3.0 × 1.7–1.9**	**Absent**	**Spider**	**This study**

##### ﻿ Gamszarella vallensis

Taxon classificationFungiHypocrealesClavicipitaceae

W.H. Chen, Y.F. Han & J.D. Liang sp. nov.

1C169DD4-7544-5A40-9ACB-9E291D55E59A

856389

Fig. 8

###### Etymology.

Referring to its location, Bala Valley, where the fungus was first discovered.

###### Type.

China • Guizhou Province, Guiyang City, Wudang District, Bala Valley (26°45'7.0344"N, 106°58'57.09"E). On a dead spider (Araneae), 5 April 2024, Wanhao Chen, GZAC WD0410 (holotype), ex-type, WD04101.

###### Description.

Colonies on PDA reaching 25–36 mm diam in 14 d at 25 °C, white, consisting of a basal felt and cottony, floccose overgrowth, reverse yellowish, with radial patterns. Prostrate hyphae smooth, septate, hyaline, 1.5–2.0 μm diam. Conidial structures consisting of erect conidiophores usually arising from the aerial hyphae, solitary or lecanicillium-like with conidiogenous cells in whorls of two to four. Conidiogenous cells 3.8–5.4 × 1.3–1.9 μm, with a cylindrical basal portion, bearing numerous denticles, tapering into a distinct neck. Conidia hyaline, ellipsoidal to fusiform, 1-celled, 2.3–3.0 × 1.7–1.9 μm. Octahedral crystals not observed.

**Figure 8.**
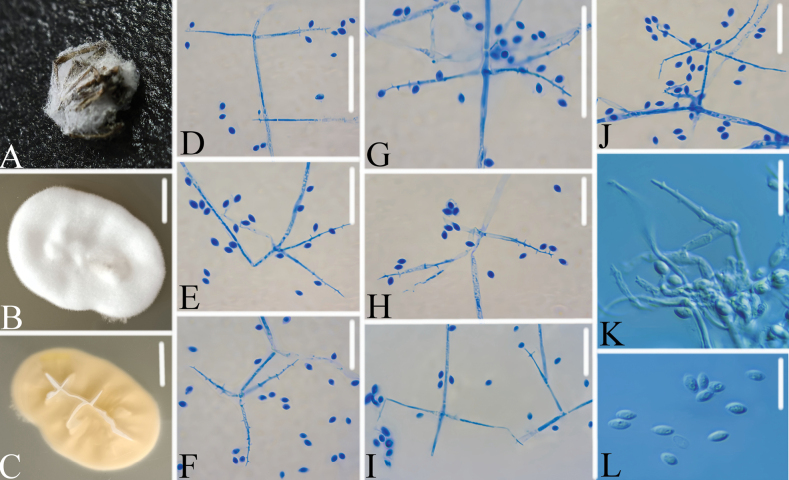
*Gamszarellavallensis***A** infected spider **B, C** pda culture plate showing top (**B**) and reverse (**C**) sides of the colony **D–J** conidiogenous cells and conidia were stained with cotton blue **K, L** conidiogenous cells and conidia. Scale bars: 10 mm (**B, C**); 10 μm (**D–L**).

###### Host.

Spider (Araneae).

###### Additional material examined.

China • Guizhou Province, Guiyang City, Wudang District, Bala Valley (26°45'7.0344"N, 106°58'57.09"E). On a dead spider (Araneae), 5 April 2024, Wanhao Chen, WD04102 (living culture).

###### Remarks.

*Gamszarellavallensis* was identified as in *Gamszarella*, based on the BLASTn results in NCBI. The phylogenetic analysis of the combined dataset 2 (Fig. 2) showed that the new strains clustered into an independent clade with a close relationship with *Gamszarellaantillana* and *G.buffelskloofina*. Compared with the typical characteristics of the known species (Table 3), *Gamszarellavallensis* was distinguished from *G.antillana* by its shorter conidiogenous cells bearing numerous denticles (3.8–5.4 × 1.3–1.9 μm vs. 18–31 × 1 μm), ellipsoidal to fusiform conidia and absent of octahedral crystals. *Gamszarellavallensis* was distinguished from *G.buffelskloofina* by its shorter conidiogenous cells (3.8–5.4 × 1.3–1.9 μm vs. 7–22 × 1.5–2 μm), smaller ellipsoidal to fusiform conidia [2.3–3.0 × 1.7–1.9 μm vs. (3–)4–6(–10) × 2 μm] and spider host. *Gamszarellavallensis* was distinguished from *G.sinensis* by its shorter conidiogenous cells (3.8–5.4 × 1.3–1.9 μm vs. 7.0–12.0 × 1.0–1.5 μm). Thus, the morphological characteristics and molecular phylogenetic results support *G.vallensis* as a new species.

##### ﻿ Neogamszarella

Taxon classificationFungiHypocrealesClavicipitaceae

W.H. Chen, Y.F. Han & J.D. Liang gen. nov.

2877EDAB-75A8-5C15-8D2D-732437846AD6

856411

###### Etymology.

Named after its morphological similarity to the genus *Gamszarella*.

###### Type species.

*Neogamszarellaantillana* (R.F. Castañeda & G.R.W. Arnold) W.H. Chen, Y.F. Han & J.D. Liang (Basionym = *Verticilliumantillanum* R.F. Castañeda & G.R.W. Arnold, Feddes Repert. Spec. Nov. Regni Veg. 98 (7–8): 411, 1987).

###### Description.

Colonies on PDA, white, with cream-colored reverse, without diffusing pigment into the agar. Conidiogenous cells developing on prostrate hyphae, single or up to 6 in verticils, subulate. Conidia solitary and of two types. Octahedral crystals present.

###### Host.

Agaric (Hymenomycetes).

###### Sexual morph.

Unknown.

###### Remarks.

The genus *Gamszarella* was established with the type species *G.buffelskloofina* and two species, *G.antillana* and *G.magnispora* (Z.F. Zhang & L. Cai) Crous based on the phylogenetic analysis (Crous et al. 2023). Khonsanit et al. (2024) introduced a new genus *Corpulentispora* Khons., Thanakitp. & Luangsa-ard to accommodate *Gamszarellamagnispora* based on the phylogenetic analysis and morphological characteristics. The morphological characteristics of *Gamszarellabuffelskloofina* are conidiogenous cells subcylindrical with apical taper, terminating in a cluster of denticles and only one type of subcylindrical conidia (Crous et al. 2023). While the morphological characteristics of *Gamszarellaantillana* are conidiogenous cells absent of denticles and produce two types of fusoid conidia, macroconidia and microconidia. *G.antillana* do not fit with the genus *Gamszarella*. Thus, a new genus is proposed to accommodate species *Gamszarellaantillana*.

##### ﻿ Neogamszarella antillana

Taxon classificationFungiHypocrealesClavicipitaceae

(R.F. Castañeda & G.R.W. Arnold) W.H. Chen, Y.F. Han & J.D. Liang comb. nov.

40E2147F-1236-579B-93FA-1795450B6AAF

856412

= Verticilliumantillanum R.F. Castañeda & G.R.W. Arnold, Feddes Repert. Spec. Nov. Regni Veg. 98 (7–8): 411, 1987. Basionym.
= Lecanicilliumantillanum (R.F. Castañeda & G.R.W. Arnold) Zare & W. Gams, Nova Hedwigia 73(1–2): 34, 2001.
= Gamszarellaantillana (R.F. Castañeda & G.R.W. Arnold) Crous, Persoonia 51: 391, 2023.

###### Remarks.

*Verticilliumantillanum* was transferred to the genus *Lecanicillium* by Zare and Gams (2001). Crous et al. (2023) introduced a new genus *Gamszarella* and combined *Lecanicilliumantillanum* with *Gamszarella* based on the phylogenetic analysis. The morphological characteristics of *Gamszarellaantillana* were similar to *Corniculantisporaaranearum* (Petch) Khons. et al., *C.dimorpha* (J.D. Chen) Khons. et al., *C.psalliotae* (Treschew) Khons. et al., *Flavocilliumacerosum* (Zare & W. Gams) H. Yu et al., and both species produce two types of conidia (Wang et al. 2020; Khonsanit et al. 2024). However, *Gamszarellaantillana* was distinguished from *Corniculantisporaaranearum*, *C.dimorpha*, *C.psalliotae* and *Flavocilliumacerosum* by the phylogenetic analysis (Fig. 2). Besides, the morphological characteristics of *Gamszarellaantillana* do not fit with the typical characteristics of the genus *Gamszarella.* Thus, *Gamszarellaantillana* was combined into the new genus *Neogamszarella* as *Neogamszarellaantillana*.

## ﻿Discussion

Karst regions in southwestern China are one of the world’s 36 biodiversity hotspots, home to a wide range of endemic species (Delgado-Baquerizo et al. 2020). Wijayawardene et al. (2021) discussed the necessity of systematic studies to reveal novel taxa in Yunnan–Guizhou Plateau. Many new entomopathogenic fungi were found in the Kasit regions of Yunnan and Guizhou Provinces (Peng et al. 2023, 2024; Tang et al. 2023a, b; Xiao et al. 2023, 2024; Zhang et al. 2023; Chen et al. 2024; Dai et al. 2024; Fan et al. 2024; Wang et al. 2024b). Besides, there is high spider diversity in the Karst regions, especially in caves (Zhang and Li 2014; Liu et al. 2023).

The present study introduces four new species of *Chlorocillium* and *Gamszarella* from spiders. *Chlorocillium* species are often found on spiders, aphids and scale insects (Zare and Gams 2016). The taxonomic delimitation of *Chlorocillium* was originally based on morphological characteristics and phylogenetic analysis of ITS or LSU sequences (Zare and Gams 2016). Tan and Shivas (2023, 2024) reported two new species based on phylogenetic analysis of ITS, LSU, *RPB2*, and *tef*-1α sequences. In this study, we introduce two new species of *Chlorocillium* viz., *C.guizhouense* and *C.vallense*, based on both morpho-molecular data (ITS, LSU and *tef*-1α) (Figs 1, 5, 6). Moreover, PHI test was carried out to visualize the differences among *Chlorocillium* species, and supports the results of morphological characteristics and phylogenetic analysis (Fig. 3). Therefore, combined analysis of morphological characteristics, phylogenetic analysis and other methods may benefit the taxonomy of *Chlorocillium*.

Crous et al. (2023) introduced *Gamszarella* with the type species *G.buffelskloofina* and transferred *Lecanicilliumantillanum* (Castañeda & G. Arold) Zare & W. Gams and *L.magnisporum* Z.F. Zhang & L. Cai into *Gamszarella* based on the phylogenetic analysis and morphological characteristics. Khonsanit et al. (2024) proposed a new genus *Corpulentispora* Khons. et al. to accommodate *Gamszarellamagnispora*. In the present study, two new species of *Gamszarella* viz., *G.sinensis* and *G.vallensis*, are introduced based on both morpho-molecular data (ITS, LSU, *RPB2* and *tef*-1α) (Figs 2, 7, 8). However, the morphological characteristics of *Gamszarellaantillana* were significantly different from *G.buffelskloofina*, *G.sinensis* and *G.vallensis* by its two types of conidia, no denticles, presence of octahedral crystals and Agaric substrate. Thus, we proposed a new genus, *Neogamszarella*, to accommodate this species.

Our study confirms the high fungal diversity associated with arthropods in South-Western China. Nevertheless, fungi associated with spiders are poorly known and need thorough, systematic exploration.

## Supplementary Material

XML Treatment for Chlorocillium guizhouense

XML Treatment for Chlorocillium vallense

XML Treatment for Gamszarella sinensis

XML Treatment for Gamszarella vallensis

XML Treatment for Neogamszarella

XML Treatment for Neogamszarella antillana

## ﻿Additional information

### Author ORCIDs

Wan-Hao Chen https://orcid.org/0000-0001-7240-6841

Dan Li https://orcid.org/0009-0009-0256-6749

Hui-Lin Shu https://orcid.org/0009-0003-1905-595X

Jian-Dong Liang https://orcid.org/0000-0002-3939-3900

Jie-Hong Zhao https://orcid.org/0000-0003-2972-382X

Wei-Yi Tian https://orcid.org/0000-0001-7365-2796

Yan-Feng Han https://orcid.org/0000-0002-8646-3975

### Data availability

All of the data that support the findings of this study are available in the main text or Supplementary Information.

## Supplementary materials

Supplementary material 1 The genera of araneogenous fungi in the order Hypocreales Data type docx https://binary.pensoft.net/file/1231129This dataset is made available under the Open Database License (http://opendatacommons.org/licenses/odbl/1.0/). The Open Database License (ODbL) is a license agreement intended to allow users to freely share, modify, and use this Dataset while maintaining this same freedom for others, provided that the original source and author(s) are credited.Wan-Hao Chen, Dan Li, Hui-Lin Shu, Jian-Dong Liang, Jie-Hong Zhao, Wei-Yi Tian, Yan-Feng Han
