# Supplementary material for: ﻿Four new araneogenous species and a new genus in Hypocreales (Clavicipitaceae, Cordycipitaceae) from the karst region of China
Source: MycoKeys. 2025 Jan 23;112:335–59. doi: 10.3897/mycokeys.112.140799 (PMC11783087; doi:10.3897/mycokeys.112.140799)
Supplement: Supplementary material 1 — The genera of araneogenous fungi in the order Hypocreales [file mycokeys-112-335-s001.docx]

Table S1 The genera of araneogenous fungi in the order Hypocreales

| Family | Genus |
| --- | --- |
| Bionectriaceae | *Clonostachys* |
| Clavicipitaceae | *Chlorocillium* |
|  | *Neoaraneomyces* |
| Cordycipitaceae | *Arachnidicola* |
|  | *Beauveria* |
|  | *Bhushaniella* |
|  | *Cordyceps* |
|  | *Corniculantispora* |
|  | *Engvodontium* |
|  | *Gamszarella* |
|  | *Gibellula* |
|  | *Hevansia* |
|  | *Lecanicillium* |
|  | *Samsoniella* |
|  | *Torrubiella* |
|  | *Jenniferia* |
|  | *Polystromomyces* |
| Ophiocordycipitaceae | *Hirsutella* |
|  | *Hymenostilbe* |
|  | *Ophiocordyceps* |
|  | *Purpureocillium* |
